# Transvenous extraction of pacemaker and implantable cardioverter defibrillator leads using Evolution® mechanical dilator sheath: a single center confirmatory experience

**DOI:** 10.1186/s40064-016-1987-x

**Published:** 2016-03-22

**Authors:** Uğur Kocabaş, Hamza Duygu, Nihan Kahya Eren, Zehra İlke Akyıldız, Serhan Özyıldırım, Selcen Yakar Tülüce, Tuncay Kırış, Cem Nazlı

**Affiliations:** Cardiology Department, İzmir Atatürk Training and Research Hospital, 35360 Basın Sitesi, Izmir, Turkey; Cardiology Department, Medical Faculty, Near East University, Nicosia, Cyprus; Cardiology Department, Farabi Hospital, Konya, Turkey

**Keywords:** Lead, Extraction, Pacemaker, Defibrillator

## Abstract

**Objective:**

In recent years there has been an increase in clinical situations requiring lead extraction procedures of implanted cardiac devices. In our clinic, extraction procedures are performed with Evolution® mechanical lead extraction system. In this manuscript we aimed to evaluate our lead extraction procedures.

**Methods:**

We retrospectively evaluated lead extraction procedures carried out on 41 patients [30 male, 11 female patient; mean age 61.5 ± 18.5 median 67 (23–85)] between 2008 and 2015 using Evolution® system. Procedural success, major and minor complications are determined according to previously published guidelines.

**Results:**

Mean duration of the lead implantation was 88.4 ± 62.5 months (6–240). Implanted device was a pacemaker in 27 (65.8 %) and ICD in 14 (34.2 %) of patients. Total 67 leads were extracted from the patients, 22 (32.8 %) were atrial, 30 (44.2 %) were ventricular, 14 (21.5 %) were dual coil defibrillator and 1 (1.5 %) was coronary sinus lead. Indications for lead removal were pacemaker decubitis and infection in 29 (70.8 %), lead dysfunction in 11 (26.8 %) and subclavian vein thrombosis in 1 (2.4 %) patient. Success rate with Evolution® system without using snare was 85.3 %. Clinical success rate was 97.5 % procedural success rate was 95.1 % and failure occured in one patient. Major complications occured in 2 (4.8 %) patients, 1 (2.4 %) was procedure related mortality. Minor complications were seen in 5 (12.2 %) of patients.

**Conclusions:**

In our single center study it is shown that extraction of pacemaker and defibrillator leads of relatively long implantation duration and in an older age patient group may be successfully carried out using the Evolution® system. However due to potentially serious complications it is adviced to be done by experienced operators in centers with cardiovascular surgery backup.

## Background

Indications of cardiac pacemaker and internal cardioverter defibrillators for management of cardiac arrhythmias and heart failure have been broadening in past decades and there has been an increase in patients with cardiac devices. Clinical problems related with implanted cardiac device and leads such as pacemaker infections, decubitis ulcers, subclavian venous thrombosis, chronic pain at implantation site necessitating device and lead removal also increased dramatically (Wilkoff et al. 2009). Leads that are implanted less than 1 year may be explanted by simple traction but as implantation duration increases, formation of fibrous capsule around the lead makes extraction procedures difficult and necessitates special devices for lead removal (Esposito et al. 2002) Mechanical extraction systems, laser and radiofrequency devices and auxiliary appereils are needed to get rid of fibrous capsule adhesions around the lead and vascular endothelium (Love 2007; Verma and Wilkoff 2004) In patients when all these methods fail, removal of leads may require open cardiac surgery.

Mechanical lead extraction systems are very useful tools developed for lead extraction procedures. They consist of specially designed locking stylets which fix the lead and a hand-powered flexible dilator plastic sheath. The sheath has threaded metal distal tip which cuts fibrous adhesions when rotated by a handle. Unlike powered sheaths like laser or electrosurgical dissection sheaths, no energy form is used for extraction. The distal steel blade of the sheath can be more effective on calcified areas on which powered sheaths are less effective (Smith and Love 2008). After the lead is fixed by locking stylet; the sheath is advanced over the lead-stylet complex to cut fibrotic adhesions and liberate the lead from vascular system. Evolution® mechanical dilator sheath which we use in our clinic for extraction procedures also has a telescopic outer polymer sheath which protects venous wall from the metal cutting tip. Leads are extracted by gentle traction, countertraction and by cutting through adhesions with this system. Mechanical extraction systems are less costly than laser systems and successful results are being reported with these systems recently (Starck et al. 2013; Mazzone et al. 2013).

In this manuscript we aimed to evaluate lead extraction procedures carried out in our clinic using Evolution® mechanical dilator sheath lead extraction system, compared results with our preliminary experience and briefly reviewed literature on lead extraction systems.

## Methods

41 Patients whose leads were extracted by Evolution® mechanical dilator sheath lead extraction system (Cook Medikal, Bloomington, IN, USA) in our clinic between 2008 and 2015 were retrospectively evaluated. Patients whose leads were explanted by simple traction without using Evolution system are excluded from the trial. The procedures were performed by the same lead extraction team of operators who are experienced in implantation and revision of pacemaker and internal cardioverter defibrillators. The lead extraction procedures using Evolution system started in our clinic in 2008 with one patient and number of our patients increased since then. Between 2009 and 2013 2, 3, 4, 4 and 6 procedures per year were done respectively. We published our preliminary experience in 2015 (Kocabas et al. 2015). When we doubled our total patient number to 41 by performing 8 procedures in 2014 and 13 in 2015 we decided to publish our confirmatory experience on lead extraction. Indications of lead extraction were determined based on Heart Rhythm Society 2009 Expert Consensus Statement on the Lead Extraction (Wilkoff et al. 2009). Informed consent forms were obtained from all patients before the procedure and local ethics committee approved our retrospective study.

### Lead extraction technique

**Fig. 1:**
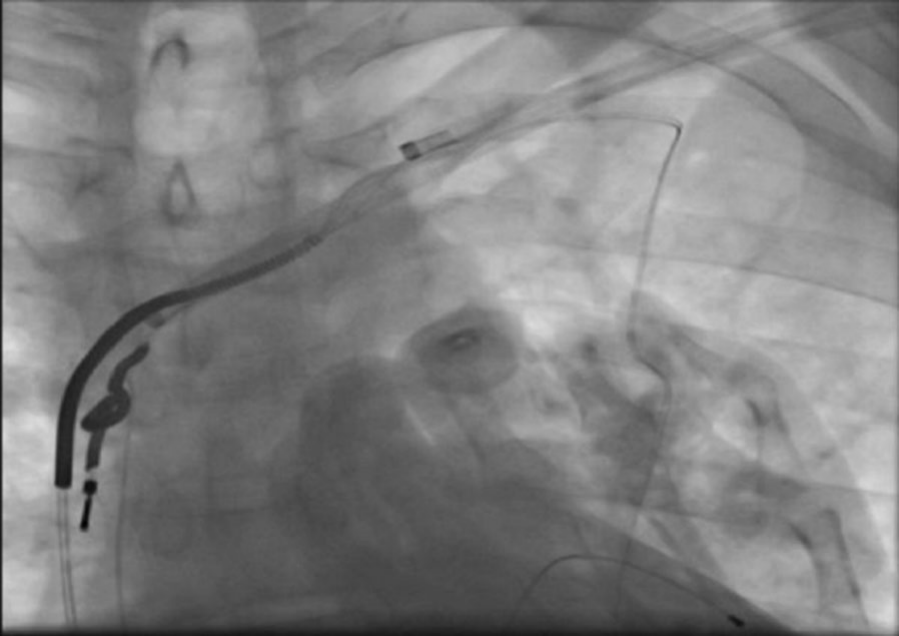
Combined use of two mechanical dilator sheaths for separation of extremely adhered leads

**Fig. 2:**
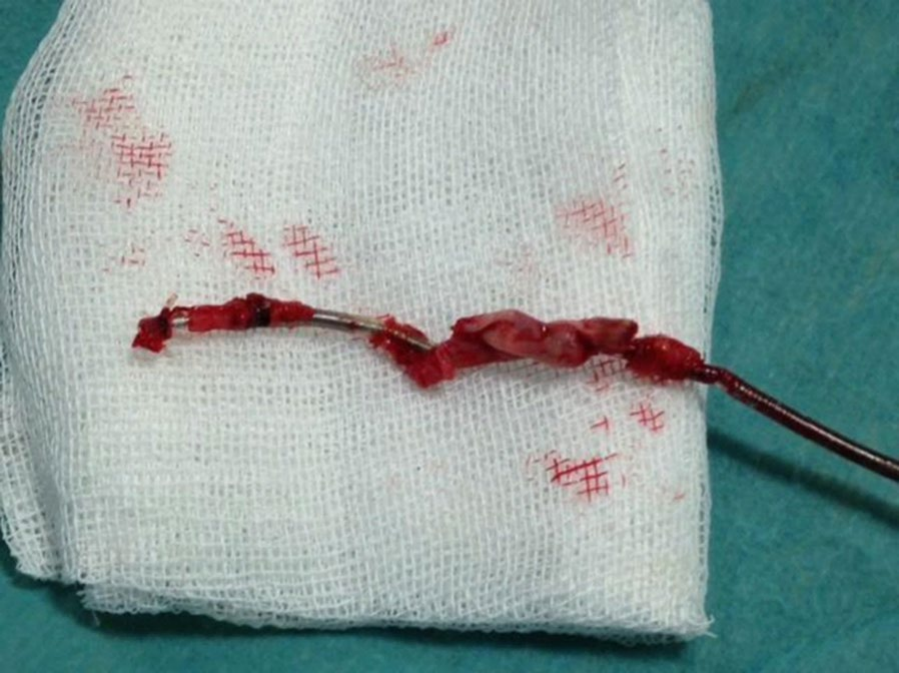
The extracted lead

Lead extraction procedures were performed in cardiac catheterisation laboratory under local anesthesia and conscious sedation with non-invasive blood pressure monitorisation. Although most experts prefer to use invasive blood pressure monitorisation during the procedure due to technical difficulties in our laboratory we used non-invasive monitorisation. We increased the frequency of blood pressure measurements at the critical stages of the procedure; during advancement of the system at the subclavian vein and superior vena cava angle and while performing countertraction at the distal tip of implanted electrodes. There are successful reports of non-invasive monitorisaton during procedure (Oto et al. 2011, 2012); but it is better to have invasive blood pressure monitorisation when available. Temporary pacemakers were implanted from femoral vein before procedure in pacemaker dependent patients. Pacemaker pocket region was explored using a sterile method and pacemaker generator was explanted. The leads were explored and separated from surrounding fibrous tissues, sleeves and the capsule using blunt dissection while protecting lead’s lumen and integrity. After dissection of the lead from fibrous tissue up to subclavian vein puncture site; patency of lead’s lumen was checked by a standard stylet. If an active fixation lead was present, it was unscrewed from the endocardium. If the lead could not be explanted by gentle simple traction then special locking sytlet was exchanged with standard sytlet. This locking stylet was advanced towards the distal endocardial implantation site to fix the lead from the distal end. If the lumen’s integrity was damaged or locking stylets could not be advanced to the distal portion of the electrodes, a bulldog system was used for the fixation of lead in 11 (26.8 %) of our patients. After the fixation of lead, mechanical dilator sheath was advanced over lead and locking stylet complex. The distal blades of mechanical dilator sheath cuts the fibrotic adhesions around the lead and the outer polymer sheath protects vascular structures. Extremely adhered leads could be separated by using two dilators and an example of such condition which occured in two of our patients is shown in Fig. 1. It was imperative to stay co-axial to the lead’s plane at all times for protecting lead’s integrity during procedure. When distal implantation site was reached, instead of dilator’s metal distal end, outer polymer sheath was advanced to perform countertraction. By countertraction the distal tip of lead was liberated from endocardium without causing avulsion. Then lead was pulled back into outer polymer sheath without leaving any foreign material in vascular system. If any lead remnants remained in vascular spaces they are removed by using snares from femoral route. We used 2 types of snares Needle’s Eye Snare (Cook Vascular, Leechburg PA USA) in one patient and Multisnare (Multisnare, PFM, Köln, Germany) in four patients. In pacemaker dependent patients who have undergone lead extraction procedure because of infection, new battery and lead implantation was performed at the contralateral site after having negative blood cultures for 72 h; which were obtained in 24 h of lead extraction procedure. In other patients the new battery and leads were implanted at the same or another session at the contralateral site. After the procedure cardiac rhythm, hemodynamic status of patient were monitored closely in the intensive care unit regarding development of bleeding complications, pericardial tamponade, arrythmia problems. A lead extracted in our hospital is shown in Fig. 2.

### Procedural success

Procedural success was defined as extraction of all leads and its components without causing any major complication. Clinical success is defined as extraction of all lead’s components or retention of a small component (<4 cm) that is not expected to cause a clinical problem in the long term. Major and minor complications are defined according to previously published guidelines.

### Statistical analysis

Statistical analysis were performed using SPSS 12.0 program. Continuous variables are expressed as mean ± SD, medians and categorical variables are defined as percentages.

## Results

41 Patients whose leads were extracted by Evolution® mechanical dilator sheath lead extraction system in our clinic between 2008 and 2015 were included in our study. The clinical characteristics, concomitant diseases and types of implanted devices are summarised in Table 1. The devices implanted to the patients and lead extraction procedure details are presented in Table 2. Results of the procedures and complications are presented in Table 3.

**Table 1:** Baseline characteristics of the study population

Age, mean ± SD	61.5 ± 18.5 median 67 (23–85)
Gender, n (%)	30 Male (73.2 %) 11 female (26.8 %)
Indication of initial device implantation, n (%)	
Sick sinus syndrome	16 (39 %)
Complete AV block	11 (26.8 %)
Ventricular arrhythmias, heart failure	14 (34.2 %)
Implanted device, n (%)	
Single chamber PM,VVI/VDD	7 (17 %), 6 (14.8 %)
Dual chamber PM	14 (34 %)
Single chamber ICD	6 (14.8 %)
Dual chamber ICD	7 (17 %)
Biventricular ICD	1 (2.4 %)
Prevalence of diseases, n (%)	
Heart failure	12 (29.2 %)
Coronary artery disease	13 (31.7 %)
Valvular disease	2 (4.8 %)
Hypertension	19 (46.3 %)
Diabetes	7 (17 %)
Congenital heart disease	3 (7.2 %)
Chronic obstructive lung disease	2 (4.8 %)
Chronic renal failure	2 (4.8 %)
Hypertrophic cardiomyopathy	1 (2.4 %)
Arrythmogenic right ventricular disease	1 (2.4 %)
Cirrohis of liver	1 (2.4 %)
Brugada syndrome	1 (2.4 %)

**Table 2:** Procedure and implanted device characteristics

Implanted device, n (%)	Pacemaker 27 (65.8 %) ICD 14 (34.2 %)
Mean duration after initial implantation	88.4 ± 62.5 months median 84 (6–240 months)
Cardiac leads per patient, total n (%)	67
One lead	17 (41.4 %)
Two leads	23(56.2 %)
Four leads	1 (2.4 %)
Lead type, n (%)	67
Atrial	22 (32.8 %)
Ventricular	30 (44.2 %)
Defibrillation coil	14 (21.5 %)
Coronary sinus lead	1 (1.5 %)
Fixation mechanism, n (%)	
Active fixation	11 (16.4 %)
Passive fixation	56 (83.6 %)
Indication for removal, n (%)	
PM pocket dekubitis, infection	29 (70.8 %)
Lead dysfunction	11 (26.8 %)
Subclavian vein thrombosis	1 (2.4 %)

**Table 3:** Procedure and procedure related complications

Clinical success, n (%)	40 (97.5 %)
Procedural success, n (%)	39 (95.1 %)
Success rate without snare use, n (%)	35 (85.3 %)
Major complications	2 (4.8 %)
Mortality after procedure	1 (2.4 %)
Right ventricular rupture caused by temporary transvenous pacemaker	1 (2.4 %)
Cardiovascular surgery due to failure of procedure	1 (2.4 %)
Minor complications	5 (12.2 %)
Vascular repair operation by vascular surgeons	2 (4.8 %)
Pacemaker pocket hematoma requiring surgery	1 (2.4 %)
Pericardial effusion	1 (2.4 %)
Pleural effusion not requiring drainage	1 (2.4 %)
Snare need for lead or its components	5 (12.2 %)
Device implantation indication after procedure	31 (75.6 %)
Patients with device implantation after procedure	25 (61.1 %)

Mean age of patients included in the study was 61.5 ± 18.5 median 67 (23–85) (Table 1). Mean duration of lead implantation were 88 ± 62.5 months, median 84 months (6–240) (Table 2). Devices were mostly implanted for sick sinus syndrome (39 %) followed by ventricular arrhythmias, heart failure (34.2 %) and complete atrioventricular block (26.8 %) (Table 1). Implanted devices were pacemakers (PM) (65.8 %) and internal cardioverter defibrillators (ICD) (34.2 %) (Table 2). Extracted leads were 44.2 % ventricular, 32.8 % atrial, 21.5 % dual coil defibrillation leads and one coronary sinus lead was present. Majority of the leads (83.6 %) had passive fixation mechanism (Table 2). In one cardiac resynchronisation defibrillator device (CRT-D) patient there was an additional nonfunctional atrial lead and in three patients there was one additional nonfunctional ventricular lead. These leads contribute to the total number of 67 leads in 41 patients. Main reason for lead extraction was device infection and decubitis (70.8 %) (Table 2).

Success rate with Evolution system only was 85.3 % and snare was used to complete the procedure in 12.2 % of patients. Procedural success was 95.1 %, clinical success was 97.5 % and failure occured in one patient (Table 3). Major complication rate was 4.8 %, one case of mortality and one thoracotomy requirement due to right ventricular rupture caused by temporary pacemaker electrode (Table 3). Our minor complication rate is 12.2 % and these were vascular repair operation in 2 patients, pacemaker pocket hematoma requiring surgery in 1 patient and pleural effusion in 1 patient and pericardial effusions in 1 patient (Table 3). Snare was used to remove retained lead components in 12.2 % of patients (Table 3). In 10 (24.4 %) of patients indication for new device implantation was not present according to the 48 h Holter recordings and 5 patients declined new device implantation so we implanted a new device to 61.1 % of total patients (Table 3). All patients who did not have indication for a new device were patients diagnosed with sick sinus syndrome previously.

## Discussion

In this single center study we demonstrated that in a patient group of advanced age and long implantation duration; pacemaker and defibrillator leads can be extracted using mechanical dilator sheath extraction system with acceptable success rates. In the literature various lead extraction methods including mechanical sheaths (polymer, steel), extraction devices (special locking stylet, snare), telescopic sheaths, mechanical extraction systems, laser and radiofrequency devices have been reported. In PLEXES study, where laser and other types of lead extraction systems (locking stylet, telescopic sheath) were used, procedural success rate were found to be 94 and 64 % for laser and other types of lead extraction systems respectively. Major complication rate was 1.96 % in laser group (Wilkoff et al. 1999). In LexIcon study laser based system had procedural success rate 96.5 % and incidence of major adverse events was 1.4 % (Wazni et al. 2010). Although laser based systems seem to have a higher procedural success rate, its procurement is very difficult and it is quite costly. Besides it is not available in our country due to social security system reimbursement policy. Also these trials compared laser system with locking stylets and telescopic sheaths rather than the Evolution® system.

Procedural success rate of lead extraction with Evolution® has been reported as 86 % by Hussein et al. (2010). Oto et al. (2011, 2012) reported their procedural success rates 82 and 87.9 % in their first and second reports respectively. Their clinical success rate was 98.5 % in second report in a patient group with mean age 55.6 ± 11.5 years and median duration of implant 85 months (22–240). Recently published studies indicate that mechanical lead extraction systems can be as safe as laser systems and may be more cost effective. In a study performed by Mazzone et al. (2013), no difference between laser and mechanical lead extraction system (Evolution®) was found with respect to procedural success (97.3 vs. 91.7 % p = 0.16), clinical success (98.6 vs. 97.9 % p = 0.76), major (2.7 vs. 4.2 %) and minor (5.5 vs. 2.7 %)complication rates. In mechanical extraction system group more frequent need for snaring lead components is observed (8.2 vs. 27.1 %). However Evolution group in this study had higher number of leads per patient (2.77 vs. 2.4 % p = 0.049) and longer implantation duration (101.1 vs. 62.4 months p = 0.01). In another study which compared both methods in patients who have longer implantation duration of mean 69.6 months, laser and mechanical extraction systems did not show any difference in procedural success rates (76.9 vs. 88.6 % vs p = 0.132). Clinical success rate (76.9 vs. 97 % p = 0.018) was better in the Evolution® group (Starck et al. 2013). Evolution® system was found superior to laser system regarding clinical success and cost effectiveness. Mechanical extraction systems are also developing their technology with innovations like short handle system for better control of the sheath and different axis rotating blade for preventing wrapping of the leads during extraction procedures and these features may result in better procedural success rates. Spectranetics® which owns laser based extraction system on the other hand developed a Tightrail™ mechanical dilator sheath system which is another mechanical extraction device similar to Evolution system. Tightrail™ sheath provides more flexibility compared to Evolution sheath and it has cutting blades inside the sheath unlike Evolution system. This feature may add safety during the extraction procedure. The preliminary clinical success rate of 95.7 % was reported by Aytemir et al. (2015) with this system. In the long term these outcomes may indicate a trend favoring mechanical systems rather than laser system. The clinical characteristics and success rates of lead extraction procedures using mechanical extraction devices reported in literature and our center’s results are summarised in Table 4

**Table 4:** Main characteristics and results from different centers using mechanical extraction devices and our center’s results

Trial	Patient number	Age of patients (years)	Mean implantation duration (months)	Clinical success rate (%)	Procedural success rate (%)	Major complication rate (%)	Minor Complication rate (%)
Hussein^a^ et al. (2010)	29	65 ± 19	65 (12–409)	100	86	0	0
Oto^a^ et al. (2011)	23	58 ± 14	74 (25–180)	100	82	0	4.3
Oto^a^ et al. (2012)	66	55 ± 11	85 (22–240)	98.5	87.9	1.5	3
Mazzone^a^ et al. (2013)	48	65 ± 14	101 ± 66.4	97.9	91.7	4.2	2.7
Starck^a^ et al. (2013)	122	60.4	69.6 (1–384)	97	88.9	2	3
Kocabas^a^ et al. (2015)	20	61 ± 19	97 (8–204)	95	95	5	25
Aytemir^b^ et al. (2015)	23	59 ± 13	72 (8–216)	100	95.7	0	0
This study^a^	41	61 ± 18	88 (6–240)	97.5	95.1	4.8	12.2

^a^Evolution mechanical lead extraction system

^b^TightRail mechanical lead extraction system

Although we can successfully remove leads with mechanical dilator sheath extraction systems, procedure has serious life threatening complications. The Evolution is a big bulky device and has to be used by at least two experienced operators one holding the lead and stylet complex and other one cutting fibrotic attachments and advancing the dilator sheath. If their manipulations are not optimally coordinated the lead’s integrity can be easily damaged and heart and great vessels may be injured during the procedure. Major complications with percutaneous lead extraction procedures are cardiac rupture (1–4 %), emergency cardiac surgery requirement (1–2 %) and death (0.4–0.8 %) (Henrikson and Brinker 2008). In our experience we had 4.8 % major and 12.2 % minor complication rates. These rates may be due to our relatively early experience, older age of patients and long implantation duration in our study. Compared to our preliminary results (Kocabas et al. 2015) clinical and procedural success rates are similar but, our minor complication rates declined and less snare use is needed with more experience with the extraction system. Learning curve mostly occurs during first 10–20 cases and when compared with our previous report need for snares 20 % and other minor complications 25 % decreased to 12.2 %. Only one additional case of snaring was required in our second half of experience and other minor complications which we reported in preliminary results did not occur. Snaring was needed mostly in patients who have passive fixation electrodes and previously damaged leads. However there was one case of mortality in our series. That patient suffered sudden cardiac arrest with electromechanical dissociation without any pericardial or pleural effusion, arrythmia or profound hypotension in intensive care unit 4 h after successful procedure. He did not respond to iv fluids vasopressors and cardiopulmoner resuscitation and expired before being taken into the cardiovascular surgery room. Autopsy was not performed but tear and late rupture of great vessels is suspected because two mechanical dilator sheaths (11F and 13F) were used in this patient to complete the extraction procedure. Experience of the operator is important in preventing complications with these extraction systems; (Smith and Love 2008) and procedure must be performed at high volume cardiovascular centers with emergency cardiovascular surgery backup.

## Conclusions

In our experience mechanical extraction systems appears to be the firstly preferred method in the extraction of leads in the long run. As number of cases performed using these systems increase their safety, effectiveness and complication rates will be understood more in detail.
